# Correction

**DOI:** 10.1111/cas.15904

**Published:** 2023-07-03

In an article^1^ titled “Let‐7b‐5p inhibits colon cancer progression by prohibiting APC ubiquitination degradation and the Wnt pathway by targeting NKD1” by Yuyang Dai, Jinsong Liu, Xuyan Li, Jianzhong Deng, Cheng Zeng, Wenbin Lu, Yongzhong Hou, Ying Sheng, Honglin Wu, Qian Liu, there were errors in the GEO dataset ID number and in Figures 5 and 10.

The correct Figures 5 and 10 are shown below:

**FIGURE 5:**
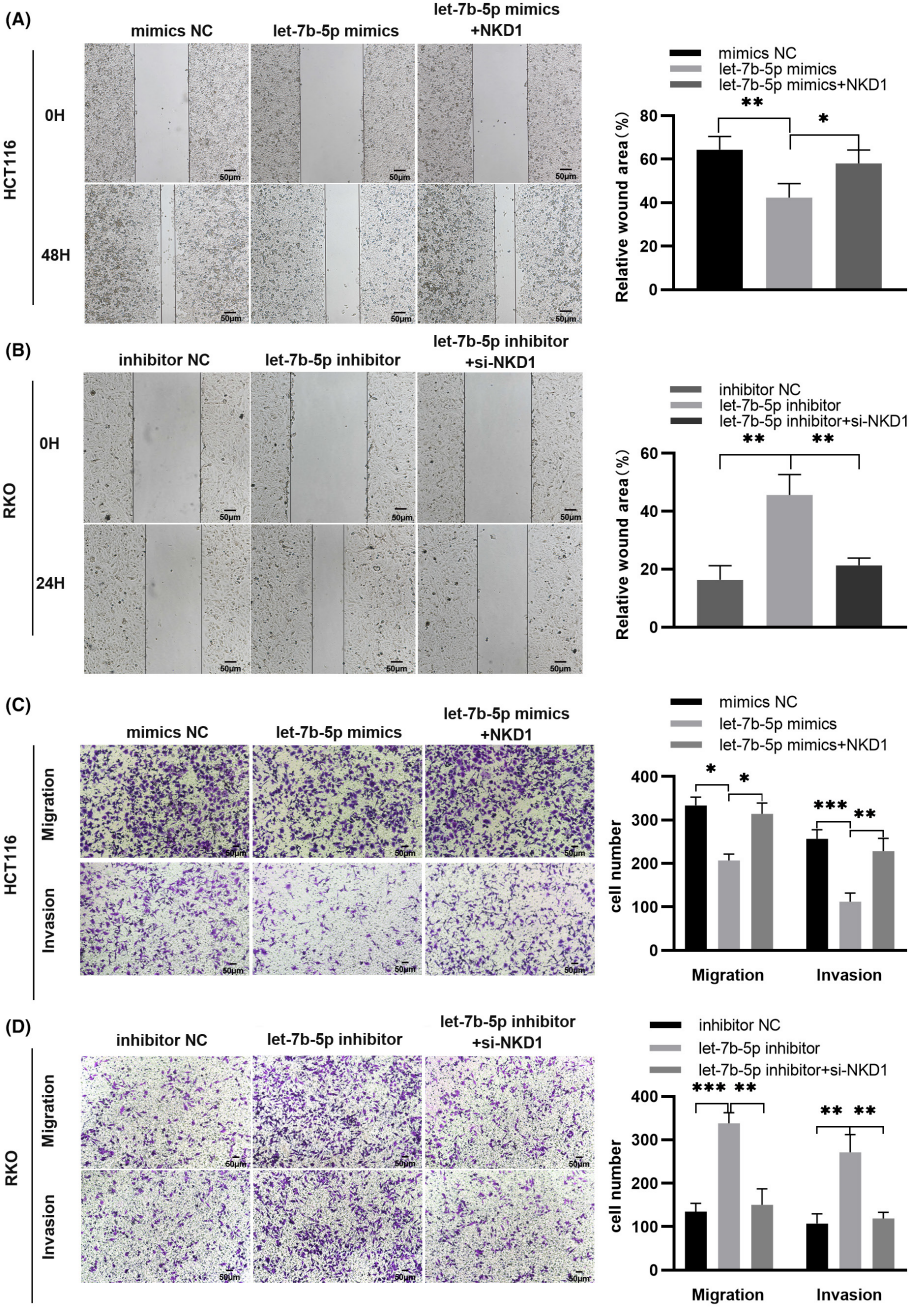


**FIGURE 10:**
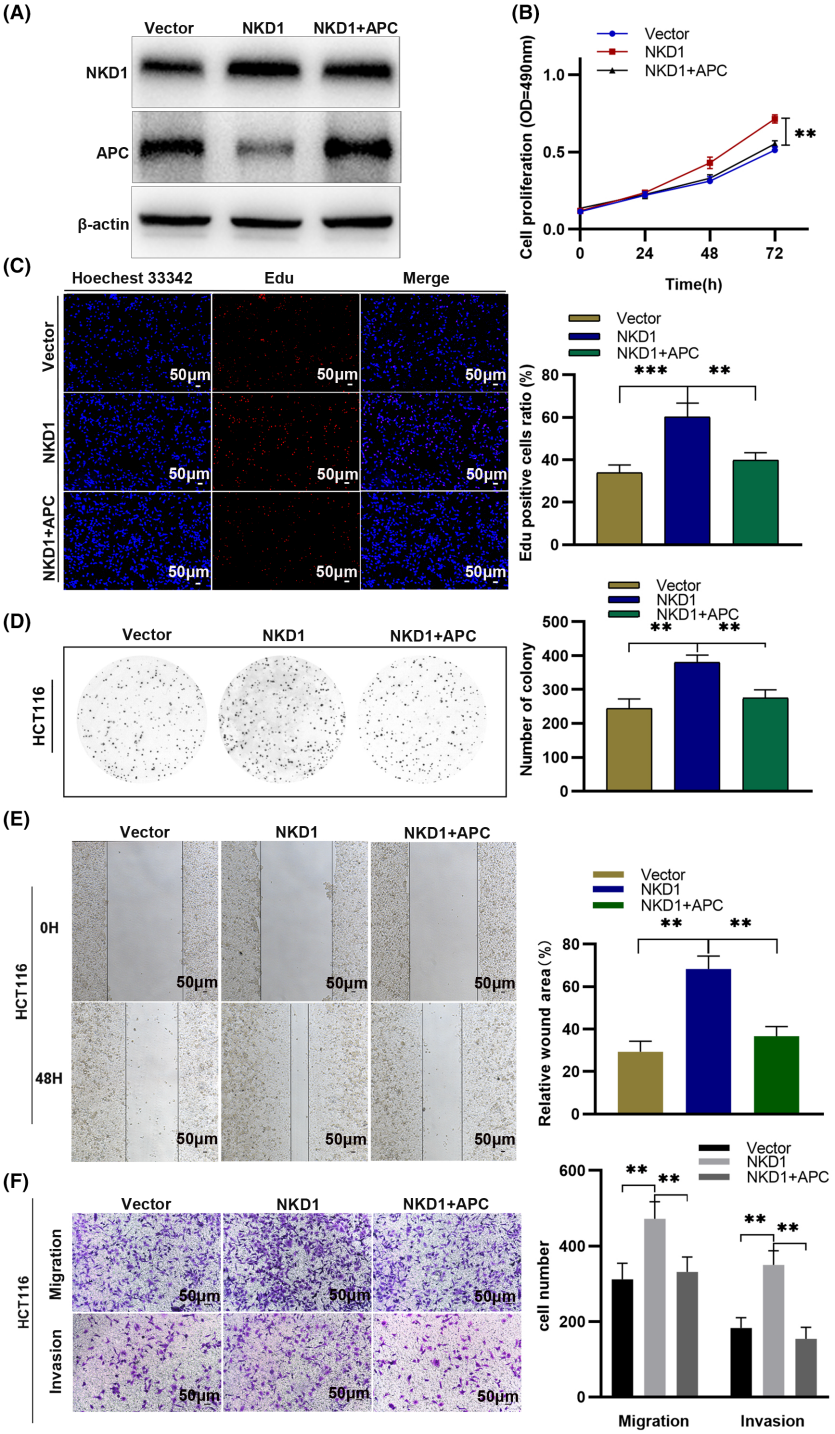


The correct GEO dataset ID number are shown below:

In Section 2.1, row 3. “The GSE135918 dataset was captured from the GEO Database”.

GSE135918 should be GSE126093.

In Section 2.2, row 2. “Screening for miRNAs linked with colon cancer phenotype, WGCNA was performed on TCGA‐miRNA and GSE195918 datasets”.

GSE195918 should be GSE126093.

In Section 2.4, row 2. “The “limma” package was employed to select differentially expressed miRNAs,17 with |LogFC>1| and FDR <0.05”.

|LogFC>1| should be |LogFC| > 1.

In Section 3.1, row 2. “Weighted gene co‐expression network analysis was performed on the TCGA‐miRNA dataset and GSE135918”.

GSE135918 should be GSE126093.

In Section 3.1, row 8. “TCGA‐miRNA data and GSE135918 were also evaluated by differential analysis”.

GSE135918 should be GSE126093.

In Section 3.1, row 11. “30 differentially expressed miRNAs were screened from GSE135918”: GSE135918 should be GSE126093.

The authors apologize for the errors.
